# *Veterinary Research* reviewer acknowledgement 2015

**DOI:** 10.1186/s13567-016-0324-2

**Published:** 2016-02-29

**Authors:** Michel Brémont, Élodie Coulamy

**Affiliations:** Unité de Virologie et Immunologie Moléculaires, Veterinary Research, INRA, Jouy-en-Josas, France

The *Veterinary Research* editorial team would sincerely like to thank all of our reviewers who contributed to peer review for the journal in 2015.

**Mohamed Abdul-Careem**

Canada

**Cristina Acin**

Spain

**Pier Luigi Acutis**

Italy

**Ben Adler**

Australia

**Judd Aiken**

Canada

**Raúl Almeida**

USA

**Rodrigo Almeida-Paes**

Brazil

**Irina Amorim**

Portugal

**Thavamathi Annamalai**

USA

**Virginia Aragon**

Spain

**Cova Arias**

USA

**Brian Austin**

UK

**María Auxiliadora Dea-Ayuela**

Spain

**Walid Azab**

Germany

**V. Balamurugan**

India

**Mitchell Balish**

USA

**Keith Ballingall**

UK

**Isabel Bandin**

Spain

**Ashley Banyard**

UK

**Paul Bartley**

UK

**Christoph Baums**

Germany

**Brad Bearson**

USA

**Karen Beemon**

USA

**Martin Beer**

Germany

**Douglas Begg**

Australia

**David Benfield**

USA

**Sven M. Bergmann**

Germany

**Paula Berguer**

Antigua and Barbuda

**Jean-Francois Bernardet**

France

**Helle Bielefeldt-Ohmann**

Australia

**Charalambos Billinis**

Greece

**Pat Blackall**

Australia

**Barbara Blacklaws**

UK

**Damer Blake**

UK

**Sandra Blome**

Germany

**Anders Bojesen**

Denmark

**Manuel Vitor Borca**

USA

**Berend-Jan Bosch**

The Netherlands

**Paolo Bosi**

Italy

**Cristina Botías**

UK

**John Boyce**

USA

**Filip Boyen**

Belgium

**Angele Breithaupt**

Germany

**Michel Brémont**

France

**Robert Briggs**

USA

**Collette Britton**

UK

**Susan Brockmeier**

USA

**Elisabetta Buommino**

Italy

**Stewart Burgess**

UK

**Christian Burvenich**

Belgium

**Luis Calvinho**

Argentina

**Julien Cappelle**

France

**Lorenzo Capucci**

Italy

**Librado Carrasco**

Spain

**Phillip Cassey**

Australia

**Joaquin Castilla**

Spain

**Elena Chaves-Pozo**

Spain

**Yahia Chebloune**

France

**Koen Chiers**

Belgium

**Pinwen Chiou**

Taiwan, ROC

**Ulas Cinar**

USA

**Edwin Claerebout**

Belgium

**Tracy Clegg**

Ireland

**Lee Cohnstaedt**

USA

**Esther Collantes Fernandez**

Spain

**Ellen Collisson**

USA

**Andrew Conlan**

UK

**Tim Connelley**

UK

**Christopher Connolly**

UK

**Brian Cooke**

Australia

**Benjamín Costas**

Portugal

**Paul Coussens**

USA

**Eric Cox**

Belgium

**Alberto Cuesta**

Spain

**John Curran**

Australia

**Marcia Dalastra Laurenti**

Brazil

**Sylvie Daminet**

Belgium

**Rohana Dassanayake**

USA

**James Daubenspeck**

USA

**Stephen Davis**

Australia

**William Davis**

USA

**Nicholas Davis-Poynter**

Australia

**Jeroen De Buck**

Canada

**Eva De Clercq**

Belgium

**Astrid De Greeff**

The Netherlands

**Henri De Greve**

Belgium

**Teresa De Los Santos**

USA

**Annemie Decostere**

Belgium

**Aldo Dekker**

The Netherlands

**Bert Devriendt**

Belgium

**Jeroen Dewulf**

Belgium

**Barbara Di Martino**

Italy

**Adama Diallo**

Austria

**Colette Dissous**

France

**Linda Dixon**

UK

**Carlos P. Dopazo**

Spain

**Fernanda Doréa**

Canada

**Michael Dove**

Australia

**Charles Martin Dozois**

Canada

**Jitender Dubey**

USA

**Richard Ducatelle**

Belgium

**Luis Enjuanes**

Spain

**Gary Entrican**

UK

**Helena Eriksson**

Sweden

**Cesar Escobedo-Bonilla**

Mexico

**Roger Evans**

UK

**Oystein Evensen**

Norway

**Pauline Ezanno**

France

**Kay Faaberg**

USA

**Ten Feizi**

UK

**Giancarlo Ferrari**

Italy

**Jaime Figueroa**

Chile

**Katja Fischer**

Australia

**U. Fischer**

Germany

**Bram Flahou**

Belgium

**Caroline Frey**

Switzerland

**Hideto Fukushi**

Japan

**Sarah Gabriël**

Belgium

**Nellie Gagne**

Canada

**Carmina Gallardo**

Spain

**Cris Gallardo-Escárate**

Chile

**Yulong Gao**

China

**José A. García-Cabrera**

Spain

**Juan J. Garrido**

Spain

**Joseba M. Garrido**

Spain

**M. Carolyn Gates**

UK

**Volker Gerdts**

Canada

**Wilhelm Gerner**

Austria

**Amanda Gibson**

UK

**Elisabetta Giuffra**

France

**Bruno Goddeeris**

Belgium

**William Golde**

USA

**Ernest A. Gould**

UK

**Luigi Gradoni**

Italy

**David Graham**

UK

**Simon Graham**

UK

**Daniel Grenier**

Canada

**Andrea Gröne**

The Netherlands

**Martin H. Groschup**

Germany

**Jean Luc Guérin**

France

**Yusong Guo**

USA

**Efrain Guzman**

UK

**Freddy Haesebrouck**

Belgium

**Katsuro Hagiwara**

Japan

**Charles Haley**

USA

**Sabine Hammer**

Austria

**John Hammond**

UK

**Josee Harel**

Canada

**David Harper**

UK

**Balazs Harrach**

Hungary

**Jiří Hejnar**

Czech Republic

**Andrew Hemphill**

Switzerland

**Tharangani Herath**

UK

**Katleen Hermans**

Belgium

**Georg Herrler**

Germany

**Michael Hess**

Austria

**Myriam Hesta**

Belgium

**Douglas Hooper**

USA

**John Hopkins**

UK

**Tiago Hori**

Canada

**Margaret Hosie**

UK

**Kurt Houf**

Belgium

**Charlie Hsu**

USA

**Falk Huettmann**

USA

**William Hughes**

UK

**Ana Hurtado**

Spain

**Gisela Hussey**

USA

**Mike Hutchings**

UK

**Antti Iivanainen**

Finland

**Thomas Inzana**

USA

**Mario Jacques**

Canada

**Mark Jenkins**

USA

**Kirsty Jensen**

UK

**Anja Joachim**

Austria

**Nick Johnson**

UK

**Clinton Jones**

USA

**Clint Jones**

USA

**Kelli Jones**

USA

**Ramon A. Juste**

Spain

**Shaden Kamhawi**

USA

**Bernd Kaspers**

Germany

**Anthony Keyburn**

Australia

**Subodh Kishore**

India

**Guilherme Klafke**

Brazil

**Doris Klingelhoefer**

Germany

**Don Klinkenberg**

The Netherlands

**Christoph Koch**

Switzerland

**Guus Koch**

The Netherlands

**Frank Koenen**

Belgium

**Wolfgang Koester**

Canada

**Ad Koets**

The Netherlands

**Michael Kogut**

USA

**Nicholas Komar**

USA

**Peter Kuhnert**

Switzerland

**Gael Kurath**

USA

**Michael Kurth**

Germany

**Andrea Ladinig**

Austria

**Jan Langeveld**

The Netherlands

**Cristina Lanzas**

USA

**Neus Latorre-Margalef**

USA

**Daniel Layton**

Australia

**Ghislaine Le Gall-Recule**

France

**Ronan Le Goffic**

France

**Jacques Le Pendu**

France

**Sandrine Lesellier**

UK

**Martin Lessard**

Canada

**Hong Li**

USA

**Yanmin Li**

USA

**Dieter Liebhart**

Austria

**Hyun Lillehoj**

USA

**John Lippolis**

USA

**Tom Lipscomb**

USA

**Annette Litster**

USA

**Ding Xiang Liu**

Singapore

**Hung-Jen Liu**

Taiwan, ROC

**Remo Lobetti**

Zambia

**Willie Loeffen**

The Netherlands

**Joel López-Meza**

Mexico

**Niels Lorenzen**

Denmark

**N. James Maclachlan**

USA

**Lone Madsen**

Denmark

**Dominiek Maes**

Belgium

**Marc Marenda**

Australia

**Marc Maresca**

France

**Raquel Marques**

Portugal

**Paolo Martelli**

Italy

**Enric Mateu**

Spain

**Mikhail Matrosovich**

Germany

**Marian Mcloughlin**

UK

**Tom McNeilly**

UK

**Kieran Meade**

Ireland

**Melha Mellata**

USA

**Xiang-Jin Meng**

USA

**Francois Meurens**

France

**John Middleton**

USA

**Simon Milling**

UK

**Rebecca Mitchell**

USA

**Adebayo Molehin**

Australia

**Maria Montoya**

UK

**Robert Moore**

Australia

**Héctor Mora Montes**

Mexico

**David Moran**

Guatemala

**Kate Mounsey**

Australia

**Joachim Mueller**

Switzerland

**Eann Munro**

UK

**Alexander Murray**

UK

**Michael Murtaugh**

USA

**K. Naeem**

Pakistan

**Venugopal Nair**

UK

**Hans Nauwynck**

Belgium

**Olivier Neyrolles**

France

**Robin Nicholas**

UK

**Tokohiko Nishizawa**

Republic Of Korea

**Pierre Nouvellet**

UK

**Niels Olesen**

Denmark

**Guilherme Oliveira**

Brazil

**Steven Olsen**

USA

**Luis Miguel Ortega-Mora**

Spain

**Angel Ortiz Pelaez**

Italy

**Pamela Osterlund**

Finland

**Romain Paillot**

UK

**Richard Paley**

UK

**Bongkyun Park**

Republic of Korea

**John Pasick**

Canada

**Nicole Pavio**

France

**Luc Peelman**

Belgium

**Andres Perez**

USA

**Elisa Pérez-Ramírez**

Spain

**Patricia Pesavento**

USA

**Wolfram Petzl**

Germany

**Maria Piccone**

USA

**Sofie Piepers**

Belgium

**Maria Pieters**

Spain

**Patrick Pithua**

USA

**A. Poli**

Italy

**Jamie Prentice**

UK

**John Prescott**

Canada

**Cinta Prieto**

Spain

**Maureen Purcell**

USA

**Qiwei Qin**

China

**Valeria Quattrocchi**

Argentina

**Pascal Rainard**

France

**Silke Rautenschlein**

Germany

**Krystle Reagan**

USA

**Patrick Redig**

USA

**Gourapura Renukaradhya**

USA

**S. Richart**

USA

**Espen Rimstad**

Norway

**Katherine Roberts**

UK

**Fernando Rodríguez**

Spain

**Alexander Rodriguez-Palacios**

USA

**Wendi Roe**

New Zealand

**Angela Römer-Oberdörfer**

Germany

**Julian Rood**

Australia

**Mirko Rossi**

Finland

**Raymond Rowland**

USA

**Charles Rupprecht**

USA

**Ivan Rychlik**

Czech Republic

**Mariana Sa E. Silva**

USA

**Armin Saalmueller**

Austria

**Yoshihiro Sakoda**

Japan

**Javier Salguero**

UK

**Oscar Daniel Salomón**

Argentina

**Siba Samal**

USA

**Yongming Sang**

USA

**Gereon Schares**

Germany

**Mariela Segura**

Canada

**Emmanuel Serrano**

Spain

**Noemi Sevilla**

Spain

**Hans-Martin Seyfert**

Germany

**I. Martin Sheldon**

UK

**Huigang Shen**

USA

**Susan Shriner**

USA

**Theodoros Sklaviadis**

Greece

**Kerstin Skovgaard**

Denmark

**Jan Šlapeta**

Australia

**Annemieke Smet**

Belgium

**Krzysztof Śmietanka**

Poland

**Hilde Smith**

The Netherlands

**Ken Smith**

UK

**Rebecca Smith**

USA

**Adrian Smith**

UK

**Daesub Song**

North Korea

**Maria Rathmann Sørensen**

Denmark

**Marcelo Soria**

Argentina

**Esteban Soto**

Saint Kitts And Nevis

**Sandra Souto**

Spain

**Simon Spencer**

UK

**Marina Spinu**

Romania

**John Spiropoulos**

UK

**Barry Stein**

USA

**Sebastian Steinfartz**

Germany

**Sophie St-Hilaire**

Canada

**Norbert Stockhofe-Zurwieden**

The Netherlands

**David Stone**

UK

**Artur Summerfield**

Switzerland

**Lotta-Riina Sundberg**

Finland

**Christina Swaggerty**

USA

**Dustin Swanson**

USA

**Raymond Sweeney**

USA

**Fumihiro Taguchi**

Japan

**Eileen Thacker**

USA

**Alexandre Thibodeau**

Canada

**Suresh Tikoo**

Canada

**Ian Tizard**

USA

**Juan Maria Torres**

Spain

**Sabine Totemeyer**

UK

**Paolo Trevisi**

Italy

**Vasilios Tsiouris**

Greece

**Chris Tuggle**

USA

**Maria Tunon**

Spain

**Kevin Tyler**

UK

**Gabriele Vaccari**

Italy

**Jean-Francois Valarcher**

Sweden

**Peter Valentin-Weigand**

Germany

**Thierry Van Den Berg**

Belgium

**Filip Van Immerseel**

Belgium

**Jeroen Van Leuken**

The Netherlands

**Alain Vanderplasschen**

Belgium

**Daisy Vanrompay**

Belgium

**Anouk Veldhuis**

The Netherlands

**Lonneke Vervelde**

UK

**Mafalda Viana**

UK

**Stefan Vilcek**

Slovakia

**Amy Vincent**

USA

**Andrea Vögtlin**

Switzerland

**Jiri Volf**

Czech Republic

**Victoriya Volkova**

USA

**Wilna Vosloo**

Australia

**Bettina Wagner**

USA

**Qihong Wang**

USA

**Hao-Ching Wang**

Taiwan, ROC

**Lingshu Wang**

USA

**Zhiliang Wang**

China

**Karin Weber**

Germany

**Neil Wedlock**

New Zealand

**Eefke Weesendorp**

The Netherlands

**Dirk Werling**

UK

**Christopher Whitehouse**

USA

**Geert Wiegertjes**

The Netherlands

**Diana Williams**

UK

**Jim Winton**

USA

**Robert Wolf**

Canada

**Guanghui Wu**

UK

**Feng Yang**

China

**Joo Mi Yi**

Republic of Korea

**Han Sang Yoo**

Republic of Korea

**Ruth Zadoks**

UK

**Huanmin Zhang**

USA

**Yan Zhang**

USA

**Yanjin Zhang**

USA

**En-Min Zhou**

China

**Stephan Zientara**

France

